# New antibody approaches to lymphoma therapy

**DOI:** 10.1186/s13045-014-0058-4

**Published:** 2014-09-09

**Authors:** Tejas Suresh, Lisa X Lee, Jitesh Joshi, Stefan K Barta

**Affiliations:** Montefiore Medical Center/Albert-Einstein College of Medicine, Bronx, NY USA; Fox Chase Cancer Center, Philadelphia, PA USA

**Keywords:** Bispecific T-cell engager, Cd-20, Pd-1, Cd-22, Monoclonal, Lymphoma, Antibodies

## Abstract

The CD20-directed monoclonal antibody rituximab established a new era in lymphoma therapy. Since then other epitopes on the lymphoma surface have been identified as potential targets for monoclonal antibodies (mAb). While most mAbs eliminate lymphoma cells mainly by antibody-dependent cellular cytotoxicity, complement-dependent cytotoxicity or direct cell death, others counter mechanisms utilized by malignant cells to evade immune surveillance. Expression of PD-L1 on malignant or stromal cells in the tumor environment for example leads to T-cell anergy. Targeting either PD-1 or PD-L1 via mAbs can indirectly eliminate cancer cells by unblocking the host intrinsic immune response. Yet another mechanism of targeted therapy with mAbs are bi-specific T-cell engagers (BiTE) such as blinatumomab, which directly engages the host immune cells. These examples highlight the broad spectrum of available therapies targeting the lymphoma surface with mAbs utilizing both passive and active immune pathways. Many of these agents have already demonstrated significant activity in clinical trials. In this review we will focus on novel CD20-directed antibodies as well as mAbs directed against newer targets like CD19, CD22, CD40, CD52 and CCR4. In addition we will review mAbs unblocking immune checkpoints and the BiTE blinatumomab. Given the success of mAbs and the expansion in active and passive immunotherapies, these agents will play an increasing role in the treatment of lymphomas.

**Electronic supplementary material:**

The online version of this article (doi:10.1186/s13045-014-0058-4) contains supplementary material, which is available to authorized users.

## Introduction

In 1997 the CD20-directed monoclonal antibody (mAb) rituximab became the first mAb approved for the treatment of lymphoma after it demonstrated significant single agent activity in indolent B-cell lymphomas [1]. Since then rituximab has become an indispensable component in the treatment of all types of B-cell Non-Hodgkin lymphomas (NHL), both alone and in combination with chemotherapeutic agents [2].

While rituximab can lead to direct cytotoxicity by induction of apoptosis, it also eliminates lymphoma cells by antibody-dependent cellular cytotoxicity (ADCC) and complement-dependent cytotoxicity [3]. Its success has spawned an immense interest in using the hosts’ immune system in selectively targeting tumor cells by attacking tumor-specific surface antigens. These surface epitopes represent ideal targets as they allow effective anticancer therapy while relatively sparing normal tissues.

mAbs represent the cornerstone of passive immunotherapy, which involves engineering of B or T cell receptors targeting a desired antigen and infusion into patients with disease. Methods to potentially increase their efficacy include conjugation of mAbs with potent cell toxin or radioisotopes, exemplified by antibody-drug conjugates (ADC) and radioimmunotherapy (RIT) respectively. Another more recent mode of passive immunotherapy is termed adoptive T-cell transfer: autologous T-cells with genetically modified T-cell receptors (chimeric antigen receptors; CARs) that specifically recognize a tumor epitope are reinfused and exert their newly acquired antilymphoma potency in the host [4]. BiTEs or bispecific T cell engagers are also examples for newer passive therapy that activates T cell destruction of lymphoma cells.

Active immunotherapy on the other hand enables the patient’s own immune system to re-engage into recognizing malignant cells which originally escaped immune surveillance. The classical example for active immunotherapy is tumor vaccines. More recently antibodies directed against CTLA4 or the PD-1/PD-L1 pathway, which unblock immune checkpoints, have demonstrated significant antitumor activity [3].

**Figure 1:**
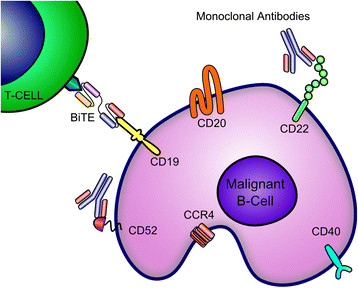
**Lymphoma cell surface targets for immunotherapy.** Abbreviations: BiTE, Bispecific T-cell Engager; CCR4, C-C Chemokine Receptor Type 4.

This review focuses on recent advances in targeting the lymphoma surface directly or indirectly with mAbs representative of active and passive immunotherapies (Figure 1), and agents that have either just reached the clinical practice or hold promise to change standard of care. Lymphoma therapy with ADCs, RIT, vaccines or adoptive T-cell transfer is reviewed elsewhere [3],[5]-[7].

### Monoclonal antibodies against B-cell antigens

#### Targeting CD20

CD20 is a surface antigen found on all mature B-cells. Its main function is to activate B-cells, allowing proliferation and differentiation. As it is also present on most mature B-cell NHL cells, it represents an ideal therapeutic target. While mAbs against CD20 target mature B-cells, they spare B cell progenitors, allowing normal B-cell regeneration [2].

Rituximab was the first mAb to target CD20 and represents a type I mAbs that cause cell death through: [8] a direct apoptotic effect; complement-dependent cytotoxicity (CDC), in which binding of the mAb activates the complement cascade; and ADCC, in which immune cells expressing Fcy receptors attack antibody-coated cells. Certain polymorphisms in the FcyRIIIa protein alter activation of effector cells causing less ADCC and result in significantly lower response rates (RR) following rituximab monotherapy [9]-[11]. Newer mAbs are being designed to better target carriers of these polymorphisms (Table 1).

**Table 1:** **Anti-CD20 monoclonal antibodies currently approved or being investigated in clinical trials for B-cell lymphomas**

mAb	Type of CD20 antibody*	Generation**	Structure	Mechanism of action (ADCC/CDC/PCD)	Comparison to rituximab	Indications tested	Phase of development	References
Rituximab	I	1	IgG1 Human Mouse chimeric	++/++/+	-	NHL/CL/DLBCL	FDA approved for NHL, CLL, DLBCL	[8]-[11]
Obinutuzumab (GA101; Gazyva™)	II	3	Murine bly-l derived humanized IgG1	++++/-/++++	Superior PCD/ADCC; no CDC	CLL/NHL	Phase 2/3 Approved in 2013 for untreated CLL in combination with CIb	[12]-[20]
Ofatumumab (HuMax-CD2O; Arzerra®)	I	1	Fully human IgG1	+++/++++/++	Superior CDC, decreased PCD	CLL/NHL	Phase 2/3 Approved for untreated CLL in combination with CIb & refractory CLL	[21]-[35]
Veltuzumab	I	2	Humanized IgG1	*++*/*++*/*+*	Longer ”off-rate”, more avid CD20 binding. can be given subcutaneously	R/R NHL/CLL	Phase 1/2	[36]-[38]
Ocrelizumab	I	2	Humanized fusion IgG1	*+++*/*+*/*+*	Enhanced binding to FCyRIIa	NHL	Phase 3	[39]
LY2469298	I	3	Modified Fc region human IgG1	*+++*/*++*/*++*	Enhanced Fc binding; superior ADCC	NHL	Phase 1/2	[40]
BM-ca	I/II***	3	Humanized IgG	*++++*/++*+*/*++++*	Different epitope, superior ACDD, CDC and PCD	NHL	Phase 1	[42]-[44]

ADCC: Antibody dependent cellular cytotoxicity; CDC: Complement dependent cytotoxicity; CIb: chlorambucil; CLL: Chronic lymphocytic leukemia; DLBCL: Diffuse large B cell lymphoma; PCD: programmed cell death; mAb: Monoclonal antibody; Moa: Mechanism of action; NHL: Non-Hodgkin lymphoma.

*Type of mAb: compared to a type I mAb, a type 2 mAb does not evoke a complement response, however, may have increased PCD/ADCC.

**Generations of mAB.

1^st^ generation: originally approved mAB against a clinically validated target 2^nd^ generation: follow-up antibodies with improved variable domains that target the same epitopes with higher or lower affinity, or have different antibody formats, e.g. Pegylation and Fc-fusion proteins. 3^rd^ generation: target different epitopes or trigger other mechanisms of action; often engineered for improved Fc-associated immune functions or half-life.

***BM-ca demonstrates properties of both Types I and II mAbs.

Obinutuzumab (GA101; Gazyva™) represents a type II mAb; while type I mAbs work primarily through CDC by stabilizing CD20 on lipid rafts, type II mAbs work mainly by direct cell death and ADCC [12]-[15]. Obinutuzumab is a glycoengineered CD20 mAb derived from the murine Bly-1 antibody [16]. Afucosylation (which increases affinity to Fc gamma receptor IIIa) of the Fc region leads to improved activation of effector cells [17]. leading to BCL-2 and caspase independent apoptosis, and hypothetically circumvents resistance [12]. As compared to rituximab, it results in increased ADCC and direct apoptosis both in vitro and in vivo [9],[17]. Type II mAbs are thought to have an advantage because type I mAbs are faced with complement-resistance factors, depletion of complement proteins [18], and bind C1q, which interferes with FcyR binding and decreases ADCC [19]. Furthermore, type II mAbs result in longer persisting anti-CD20 mAb complexes [20] and higher binding affinity thereby increasing ADCC.

In November 2013, obinutuzumab was FDA approved for the treatment of previously untreated CLL in combination with chlorambucil (Cb). In a phase 3 study in treatment naïve elderly patients, Cb with obinutuzumab showed superior RR and progression free survival [PFS] compared to Cb alone and Cb with rituximab (complete response [CR] rate 21%; overall response rate [ORR] 78%) [21]. In addition, obinutuzumab has been tested in combination with other chemotherapeutic agents in CLL [22] and more aggressive B-cell NHL, such as diffuse large B-cell lymphoma (DLBCL) and mantle cell lymphoma (MCL) [23], demonstrating promising results. The main non-hematologic side effects (SE) were grade 1 or 2 infusion-related reactions (IRRs) and the most common hematologic SE was neutropenia.

### Ofatumumab

Ofatumumab (HuMax-CD20; Arzerra®) is another humanized CD20-directed mAb. It binds to both loop domains of CD20 at a different epitope than rituximab and induces CDC [24]. As compared with rituximab and obinutuzumab, ofatumumab results in the greatest complement activation and antibody-dependent phagocytosis (ADP) [25].

Ofatumumab is FDA-approved in combination with chlorambucil for the treatment of CLL patients for whom fludarabine-based therapy is considered inappropriate [26] and those who are refractory to fludarabine and alemtuzumab [27]. The most common SE were IRRs and infections that were grade I/II events. Additionally, in combination with pentostatin and cyclophosphamide it compared favorably to historical controls treated with fludarabine, cyclophosphamide and rituximab (FCR) [28]-[30]. When combined with fludarabine and cyclophosphamide (O-FC) the results were comparable to what has been reported with other similar chemoimmunotherapy (CIT) regimens [31]. Trials directly comparing rituximab-based CIT to ofatumumab-based CIT in CLL are currently ongoing.

Ofatumumab has also been tested in indolent and aggressive NHL either as single agent or in combination with chemotherapy [32]-[35]. It appears that while the toxicities are similar to rituximab-based therapy the efficacy compares favorably.

### Veltuzumab

Veltuzumab is a humanized anti-CD20 mAb that was constructed on the framework regions of the anti-CD22 mAb epratuzumab (see below). Structurally it differs from rituximab by only one amino acid. It has a significantly higher potency than rituximab in preclinical models, exhibiting a greater CDC and possessing a slower off-rate resulting in longer cell surface retention [36].

In a phase 1/2 study of 82 patients with refractory NHL, the drug was well tolerated, with no serious side effects. In patients with follicular lymphoma (FL) who had prior exposure to rituximab, veltuzumab was associated with an ORR of 44% and a CR rate of 27% [37]. RR were higher in rituximab-naïve patients (ORR 57%; CR/CRu (unconfirmed CR) rate 43%). Among non-follicular histologies, the ORR was 35%, with 27% achieving a CR. Although developed for IV use, veltuzumab has been shown to have similar efficacy as a SQ injection [38].

### Ocrelizumab

Ocrelizumab is another humanized IgG1 anti-CD20 mAb. It differs from rituximab at the complementarity-determining regions, and is derived from a different allotype of human Fc. Like rituximab, ocrelizumab works through ADCC, CDC and apoptosis, although it has been shown to have better ADCC and lower CDC. Importantly, ocrelizumab has better binding to the low-affinity variants of the Fcy receptor IIIa. Patients with the high affinity variant of FcyRIIIa have shown superior outcomes following rituximab compared with patients with the low affinity variant; thus it is hypothesized that ocrelizumab may have better clinical efficacy [9],[11]. In a phase 1/2 trial, ocrelizumab was tested as single agent in patients with relapsed/refractory (R/R) FL [39]. Overall, the drug was well tolerated, (a similar safety profile as rituximab monotherapy) with an ORR of 38%, which is comparable to rituximab re-treatment.

### LY2469298

LY2469298 (AME-133v) is a humanized IgG1 anti-CD20 mAb with a 13–20 fold higher affinity to CD20 than rituximab. A limited number of amino acid substitutions in the Fc region of the mAb result in enhanced ADCC (6-fold more potent in vitro) but with 50% less CDC compared with rituximab [40]. and potentially more efficacy than rituximab in those patients who were carriers of the low affinity FcyRIIIa allele. In a phase 1 trial of patients with previously treated FL who were FCyRIIIa carriers, the drug was well tolerated; responses (PR or CR) were observed in 22% of patients [41]. In a Japanese phase 1 study the ORR was 50% in previously rituximab-treated FL patients carrying the FCyRIIIa variant [40].

### BM-ca

BM-ca is a novel mAb targeting CD20 that recognizes a unique epitope as compared to rituximab, and was stronger than rituximab in ADCC and direct anti-cell proliferation assays [42],[43]. In phase I studies, it was shown to be well tolerated with promising preliminary anti-lymphoma activity in B cell NHL (2 CR and 2 PR out of 12 patients) [44].

### Targeting CD22

CD22 is a sialic acid-binding immunoglobulin (Ig)-like lectin involved in cellular adhesion, regulation of B-cell homing and modulation of B-cell activation [45]. It is expressed by pre-B, mature, and normal B-cells as well as in many malignant B-lymphocytes [46]. During early B-cell development it is found in the cytoplasm, then on the cell surface of mature B-cells [47]. Quickly internalized when bound by mAbs, it is then re-expressed on the cell membrane after modulation, a property not found in CD20 [48],[49]. This, and the role CD22 plays in B-cell signaling, makes it an ideal target in lymphoid B-cell malignancies (Table 2).

**Table 2:** **Monoclonal antibodies directed against non-CD20 surface epitopes**

mAB	Target	Structure	Unique properties	Indication tested	Phase of development	References
Epratuzumab	CD22	Humanized IgG1	Enhanced ADCC, no PCD	NHL/DLBCL	Phase 1/2	[50]-[56]
Medi 551	CDI9	Afucosylated humanized IgG1	Enhanced ADCC, no CDC	NHL/DLBCL/ALL	Phase 1/2	[60],[61]
Lucatumumab	CD4O	Humanized IgG1	Also being tested in myeloma. ADCC	NHL/CLL/HL/MZL/MALT	Phase 1/2	[64],[69],[70]
Dacetuzumab	CD4O	Humanized IgG1	Partial agonist. Enhanced ADCC/CDC/PCD	DLBCL	Phase 1	[71]-[76]
Alemtuzumab (Campath®)	CD 52	Humanized IgG1	Enhanced ADCC/CDC/PCD	CLL/T-Cell NHL Approved for del17p CLL	Phase 2/3	[81]-[89]
Mogalizumab	CCR4	Afucosylated humanized IgG1	Enhanced ADCC	PTCL/CTCL	Phase 1/2	[91]-[98]
Pidilizumab (CT-011)	PD-1*	Humanized lgG1 Kappa recombinant	Reverses T-cell anergy	NHL/DLBCL	Phase 1/2	[101]-[113]
Blinatumomab	BiTE (CD3/CD19)	Single-chain bispecific antibody construct with variable regions of two antibodies.	Bispecific T-cell Engager	DLBCL/ALL	Phase 2/3	[118]-[120]

ADCC: Antibody dependent cellular cytotoxicity; ALL: Acute lymphoblastic leukemia; BiTE: Bispecific T-cell Engager; CDC: Complement dependent cytotoxicity; CLL: Chronic lymphocytic leukemia; CTCL: Cutaneous T Cell Lymphoma; DLBCL: Diffuse large B cell lymphoma; HL: Hodgkin lymphoma; mAB: Monoclonal antibody; MALT: Mucosa associated Lymphoid tissue lymphoma; Moa: Mechanism of action; MZL: Marginal zone lymphoma; NHL: Non Hodgkin lymphoma; PCD: programmed cell death; PTCL: Peripheral T Cell Lymphoma.

*PD1 is mainly expressed on regulatory T cell surface, while its ligand PDL-1 is often expressed on malignant cells. Stimulation of this pathway results in immune anergy towards malignant cells.

### Epratuzumab

The mAb targeting CD22 furthest along in development is the IgG1 humanized mAb, epratuzumab. The actual mechanism of epratuzumab has not been formally explored, but it is reasonable to assume that it includes ADCC, CDC and direct cytotoxicity [50]. Single agent epratuzumab has been studied in indolent as well as aggressive NHL. In an early phase 1/2 trial, epratuzumab was well tolerated and showed the best response in FL (ORR 24%) [51], while 15% of patients with DLBCL showed a response [52]. The drug was very well tolerated, with no dose-limiting toxicity.

Epratuzumab plus rituximab has been tested in R/R NHL and compared to single agent use, resulted in a higher ORR of 47% with the highest RR again in FL (64%) [53]. Another multicenter trial showed an ORR of 54% for patients with FL and 57% for small lymphocytic lymphoma (SLL) [54]. The combination of epratuzumab with rituximab was also studied in patients with newly diagnosed FL, and the RR of was 88.2% [55].

In aggressive lymphomas, when combined with R-CHOP for patients with DLBCL, the ORR was 96% [50], which compares favorably with studies using R-CHOP for upfront treatment. Of note, approximately 15% of patients with DLBCL do not express CD22; in this trial CD22-negative patients were ineligible [56].

### Targeting CD19

CD 19 is a transmembrane glycoprotein that is expressed by normal and malignant B-cells from early pre-B maturation to terminal plasma cell differentiation [57],[58]. It is found on a wide range of B-cell malignancies, including those arising from early B-cell precursors, which cannot be effectively targeted with CD20 Abs [57]. Like CD22, but unlike CD20, it is also efficiently internalized. Its function encompasses regulating cell signaling thresholds and serving as a co-stimulatory molecule for B-cell receptor (BCR) signaling [59].

### MEDI-551

MEDI-551 is an afucosylated anti-human CD19 mAb with in vitro and in vivo activity against lymphoma [60]. Results from a phase 1 trial of single agent MEDI-551 in R/R B-cell malignancies show an acceptable safety profile and ORR of 24%, 24%, and 31% in heavily pre-treated CLL, DLBCL and FL patients respectively [61]. Phase 2 trials in DLBCL patients are currently recruiting.

### Targeting CD40

CD40 is a type-1 transmembrane protein and expressed in more than 90% of B-cell malignancies [62]-[65]. It is thought to have a greater range of expression than CD20 and is present in the pro-B to the plasma cell phase of B-cell development. Studies have showed that activation of CD40 results in enhanced survival of neoplastic B-cells, thus targeting CD40 with mAbs could help block this [64]. Additionally, CD40 signaling impacts resistance mechanisms to chemotherapy. In CLL, CD40 activation triggers phosphorylation of ERK1/2 and IKK, and up-regulates Mcl-1 and Bcl-xl, which creates a malignant phenotype [64]. Similar mechanisms have been shown in Hodgkin lymphoma (HL) [66]. The prognostic significance of CD40 expression on lymphoma cells [67] and/or the bone marrow stromal cells [68] as well as the impact of CD40-related BCR signaling are areas of ongoing investigation.

### Lucatumumab

Lucatumumab, a human anti-CD40 mAb, was shown to cause more B-cell lysis than rituximab in preclinical models [64]. In a phase 1 trial in CLL, stable disease (SD) was observed in 17 of 26 patients [69]. In another phase 1/2 trial of 111 patients with R/R NHL or HL, the drug was well tolerated with ORR of 33% in FL patients and 11% in those with DLBCL and marginal zone lymphoma (MZ) [70].

### Dacetuzumab

Dacetuzumab is another CD40 mAb that acts as a partial agonist at the CD40 receptor [71]. It works through direct signal transduction, ADCC and ADP [71]. In lymphoma xenograft models it demonstrates synergy with rituximab and gemcitabine [72]. Dacetuzumab monotherapy appears to be well tolerated and without major adverse events (AEs) [73],[74]. When combined with rituximab and gemcitabine for elderly patients with R/R DLBCL (n = 33) 47% achieved a response (20% CR) [75]. These results are comparable to the efficacy of R-GemOx in the 2^nd^- line setting for DLBCL [76].

### Targeting CD 52

The CD52 antigen is a cell surface glycoprotein of unknown function that is expressed on both B- and T-lymphocytes [77]. It is recognized by a humanized mAb named alemtuzumab, which works by complement-induced cell lysis, direct cell-mediated cytotoxicity and induction of apoptosis [78]-[80].

### Alemtuzumab

Alemtuzumab (Campath®) first received accelerated approval in the U.S. in 2001 for CLL patients who had failed fludarabine. Then, based on the results of a trial comparing alemtuzumab to chlorambucil as 1st-line treatment, it received full approval in 2007 in the U.S. and 2008 in Europe [81],[82]. The subgroups that appeared to benefit the most included patients with 17p deletion, bone marrow infiltration and refractory autoimmune cytopenia [83]. In T-cell lymphomas (TCL), alemtuzumab has shown efficacy as a single agent and in combination with conventional chemotherapy in R/R or untreated peripheral TCL (PTCL) as well as in advanced cutaneous TCL (CTCL) [84]-[86].

More recent trials looked at improving the safety profile of alemtuzumab, and its effectiveness in combination with other regimens. Previous trials with alemtuzumab had been associated with significant toxicity, stemming mainly from profound immunosuppression. Lower doses of alemtuzumab showed similar effectiveness with a better safety profile [87]. Subcutaneous alemtuzumab in combination with rituximab in fludarabine-refractory CLL patients was also well-tolerated and allowed patients to achieve adequate cytoreduction prior to stem cell transplantation [88]. A recent phase 2 trial tested alemtuzumab consolidation after CHO(E)P-14 in 41 patients with untreated PTCL [89]. While the combination was quite effective (59% of patients achieved a CR), it was associated with significant treatment-related adverse events (the main grade ¾ toxicities were infections and neutropenia, including one potentially treatment-related death). Therefore, although alemtuzumab is an active drug in lymphomas, its use has been limited by its toxicities.

### Targeting CCR4

The chemokine receptor CCR4 is expressed on a subset of Type 2 helper (T_H_) and regulatory T-cells (Treg) and is involved in lymphocyte trafficking. Many adult PTCL express both CCR4 and its ligands. CCR4 (+) T-cell lymphomas are associated with a poorer prognosis, possibly because of downregulation of T-cell mediated antitumor host response [90]. Mogamulizumab (KW–0761) is a mAb that targets CCR4(+) tumor cells by ADCC and downregulates Treg trafficking to the tumor microenvironment.

### Mogamulizumab

Preliminary data shows responses in a subset of T-cell lymphomas with traditionally poor prognosis. In a phase 1 trial of 16 patients with R/R CCR4(+) mature T-cell lymphomas, 31% (n = 5) achieved a response (CR: 13%; n = 2) [91]. Results of a phase 2 trial in 28 patients with R/R CCR4(+) adult T-cell leukemia/lymphoma (ATLL) showed an ORR of 50%, a median PFS of 5.2 months and OS of 13.7 months, which lead to its approval in Japan for this indication [92]. A US trial of single agent mogamulizumab in patients with both CCR4(+) and CCR4(−) R/R CTCL (n = 38) demonstrated an ORR of 35% [93]. In a consecutive study in patients with CCR4(+) PTCL or CTCL (n = 38), the ORR was 35% (n = 13) and 14% (n =5) showed a CR with a median PFS of 3 months [94]. Infusion reactions were common (59%), but only 2% were grade III or higher. Skin and subcutaneous tissue disorders occurred in 50% of patients with 12% being grade III or higher. Viral reactivation, lymphopenia, and neutropenia were other notable AEs.

While CCR4 mAbs have primarily been studied in T-cell NHL, it has been hypothesized that influencing the tumor microenvironment by halting Treg trafficking through CCR4 blockade may be broadly beneficial in many cancers [95]-[98].

### mAbs unblocking immune checkpoints

While most mAbs in this category only indirectly target the lymphoma surface, they are included in this review as they exemplify the concept of active immunotherapy.

### PD-1/PD-L1 pathway

Programmed cell death 1 (PD-1) is a negative costimulatory receptor critical for the suppression of T-cell activation. It is part of an immunoglobulin superfamily (B7) and expressed on T- and B-lymphocytes, natural killer (NK) cells, monocytes, and dendritic cells [99]. There are two PD-1 ligands: PD-1 ligand 1 (PD-L1/B7-H1) and PD-L2/B7-DC. The expression of PD-1 is significantly increased on CD4+ and CD8+ T cells following chronic exposure and stimulation with antigens related to infection or tumors [100].

**Figure 2:**
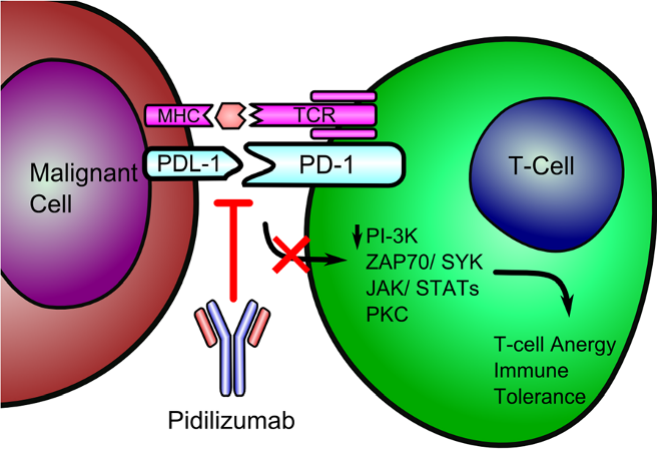
**Mechanism of pidilizumab, which increases T cell activation and cytokine release by inhibiting co-inhibitory signaling up-regulated by tumors.** Abbreviations: MHC Major Histocompatibility Complex; TCR, T-cell Receptor; PDL-1, Programmed Death Ligand 1; PD-1, Programmed Cell Death Protein 1.

On binding to its ligand, PD-1 generates a TCR–PD-1 microcluster [101], decreasing the phosphorylation of the multiple downstream signaling molecules (including Zap70, PI3K, and PKC-θ [102]) by recruiting SHP2, which in turn results in the attenuation of T-cell activation and so called “T-cell exhaustion”. Blockade of the PDL-1/PD-L2 and PD-1 interaction was shown to render previously anergic T-cells responsive to antigen [103] (Figure 2).

Infiltration of anergic PD-1 positive T-cells has been demonstrated in lymphomas [104]. PD-L1 expression can be shown in a variety of B- and T-cell lymphomas [105]-[108]. Additionally, expression of PD-1 peripheral blood CD4+ and CD8+ lymphocytes has been described as markedly elevated in patients with lymphomas, including T-cell NHL, especially at the time of relapse [109].

### Pidilizumab

Pidilizumab (formerly CT-011) is a humanized IgG-1κ recombinant mAb that targets PD1. A phase 1 trial conducted by Berger et al. [110] enrolled 17 patients with advanced hematological malignancies including acute myeloid leukemia (AML), CLL, NHL, HL and multiple myeloma (MM). It concluded that CT-011 was safe and well tolerated, with clinical benefit observed in 33%.

This was followed by a phase 2 international trial studying patients with DLBCL, primary mediastinal B-cell NHL or transformed indolent NHL, undergoing autologous stem cell transplant (ASCT) [111]. Patients received pidilizumab for three cycles, beginning 30 to 90 days after their ASCT. Among the 66 eligible patients, 16-month PFS was 72% while 16-month OS was 85%. No severe unexpected toxicities, significant autoimmune toxicities or treatment-related mortality was observed.

Another phase 2 study explored the efficacy of PD-1 blockade in combination with rituximab in relapsed rituximab-sensitive FL (n = 30) [112]. Pidilizumab was dosed every 4 weeks times four (additional doses for patients with SD or better) with weekly rituximab infusions times 4. Of 29 patients evaluable for activity, 19 (66%) achieved an objective response. CR was identified in 15 (52%) and PR in 4 (14%) patients; median PFS was 18.8 months. The combination was well tolerated, with no severe autoimmune or treatment-related AEs.

Other mAbs targeting PD-1 or PD-L1 directly are under investigation. While it appears that PD-L1 expression on tumor cells is a necessary prerequisit [113], further research is needed to identify subsets of patients who most likely benefit from blockade of this axis. Potential biomarkers of response are tumor infiltrating lymphocytes, certain T-effector cell gene signatures or increased expression of PDL-1 in circulatory leucocytes [112].

Like PD-1, CTLA-4 is a negative regulator of T-cell activation that serves to dampen antitumor immune responses. Its ligand, B7-1, is found on APCs, B-cells and certain tumor cells. Blockade of CTLA-4 has yielded increased T-cell mediated anti-tumor responses, most notably in metastatic melanoma [114]. Ipilimumab (Yervoy®), a CTLA-4 mAb, has been approved for treatment of metastatic melanoma. In a phase 1 trial ipilimumab was used to treat 18 patients with R/R DLBCL [115]. Responses were seen in 2 patients (1 with a CR lasting >31 months) and the drug was generally well tolerated, with diarrhea and fatigue as the only severe AE. Larger studies are ongoing to further explore the use of CTLA-4 blockade in hematological malignancies.

Unusual toxicities are a concern when unblocking immune checkpoints. Even though preliminary studies indicate that pidilizimab is well tolerated, studies involving other PD-1 inhibitors (e.g. nivolumab) and CTLA-4 mAbs have reported a myriad of AEs, including 3 treatment-related deaths reported with the use of nivolumab due to pneumonitis. Common AEs include autoimmune disorders such as endocrinopathies (e.g. hypophysitis, hypothyroidism), skin disorders (e.g. rash, vitiligo), pneumonitis and colitis [116].

### Bispecific T-cell engagers (BiTE)

**Figure 3:**
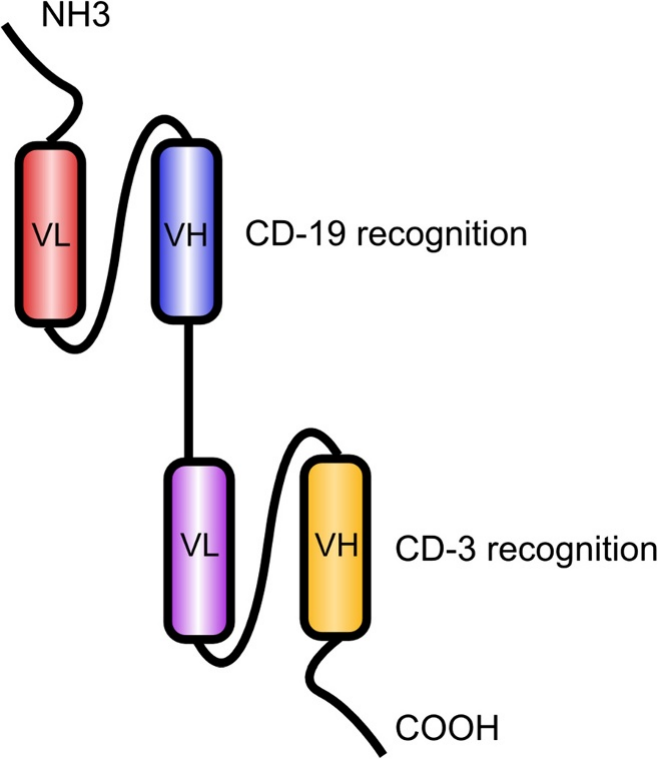
**The bispecific T-cell engager blinatumomab targeting CD-19.** Abbreviations: VL: variable region light chain; VH: variable region heavy chain.

BiTE molecules are engineered to contain the variable domains of two antibodies joined together: one antibody binds CD19 and one binds the CD3 antigen of T-cells. When bound to a CD3/CD19 complex, a BiTE brings the two cells in close proximity and thus activates T-cells to destroy the tumor cell via perforin-mediated apoptosis (Figure 3) [117].

### Blinatumomab

Blinatumomab is a BiTE molecule that has been the forerunner of BiTE molecule testing and stands for B-lineage anti-tumoral mAb. Promising activity has been demonstrated in patients with B-lineage ALL, specifically in MRD eradication [118]-[120].

The first phase 1 trial of blinatumomab as single agent given as continuous intravenous infusion in NHL began in 2004. The initial cohort of 38 patients had R/R B-cell NHL and received a continuous infusion at different doses for 4-8 weeks. Eleven patients (28.9%) had measurable response after treatment; 4 (11%) achieved a CR and 7 (18%) a PR [121]. The trial established the maximum tolerated dose (MTD) of 60 μg/m^2^/d. By 2011, the study had enrolled 62 patients. Of the 22 patients who received the MTD, 18 (82%) showed an objective response and duration of response lasted up to 32 months.

Because of its clinical benefits and tolerability in indolent lymphomas, the study was expanded to include patients with DLBCL [122]. Twelve patients were enrolled with 9 patients evaluable for response. Five out of 9 patients (56%) showed responses, the longest lasting 428 days. This set the stage for a phase 2 trial of blinatumomab in R/R DLBCL. Of the 11 patients recruited so far, 7 were evaluable for response: 3 patients experienced progression of disease, while 4 responded resulting in an ORR of 57% [120].

The most common clinical AEs regardless of grade included pyrexia, fatigue, headache, diarrhea, and weight increase. The dose-limiting factor was CNS related toxicity ranging from tremor, disorientation, speech disorder, cerebellar symptoms, to seizures.

While the results are intriguing, the optimal setting for blinatumomab in lymphomas remains to be defined. Multiple trials studying blinatumomab in B-cell malignancies are ongoing, the focus being B-lineage ALL.

## Conclusion

Tremendous advances have been made in targeting the lymphoma surface. Initially only seen as a way to more precisely target tumors, actively harnessing the ability of the patients’ own immune system in the fight against cancer is revolutionizing therapy. This involves rethinking current treatment paradigms in terms of response assessment [123] and side effect management. Unleashing the immune system can result in never-before encountered side effects. While results are promising, one remaining challenge is to identify which patient will respond to immunotherapy. Nevertheless, next to the classical modalities surgery, radiation, chemotherapy, and more recently molecularly targeted therapies, many regard immunotherapy now as the fifth pillar of oncology [124].

## Authors’ information

SKB is an assistant professor of medicine and part of the lymphoma team at Fox Chase Cancer Center.

## Authors’ original submitted files for images

Below are the links to the authors’ original submitted files for images. Authors’ original file for figure 1 Authors’ original file for figure 2 Authors’ original file for figure 3

## Abbreviations

mAB: Monoclonal antibodies
NHL: Non Hodgkin’s lymphoma
ADCC: Antibody dependent cellular cytotoxicity
ADC: Antibody drug conjugates
RIT: Radioimmunotherapy
CaRs: Chimeric antigen receptors
BiTEs: Bispecific t-cell engagers
CDC: Complement dependent cytotoxicity
RR: Response rates
PFS: Progression free survival
CR: Complete response
ORR: Overall response rate
DLBCL: Diffuse large b-cell lymphoma
MCL: Mantle cell lymphoma
ADP: Antibody dependent phagocytosis
FCR: Fludaribine + cyclophosphamide + rituximab
O-FC: Fludarabine + cyclophosphamide
CIT: Chemoimmunotherapy
FL: Follicular lymphoma
R/R: Relapsed/refractory
Ig: Immunoglobulin
SLL: Small lymphocytic lymphoma
BCR: B-cell receptor
HL: Hodgkin’s lymphoma
SD: Stable disease
MZ: Marginal zone lymphoma
TCL: T cell lymphomas
PTCL: Peripheral t cell lymphoma
TCL (CTCL): Advanced cutaneous
Treg: Regulatory t cells
ATLL: Adult T cell leukemia/lymphoma
PD-1: Programmed cell death 1
NK: Natural killer
AML: Acute myeloid leukemia
MM: Multiple myeloma
ASCT: Autologous stem cell transplant
