# Supplementary figures and images for: New antibody approaches to lymphoma therapy
Source: J Hematol Oncol. 2014 Sep 9;7:58. doi: 10.1186/s13045-014-0058-4 (PMC4172963; doi:10.1186/s13045-014-0058-4)

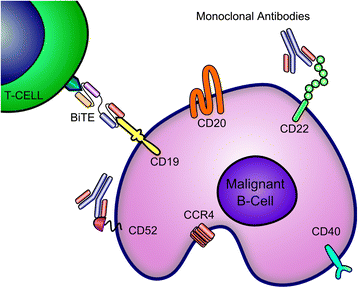

Supplement: Supplementary file 1 — Authors’ original file for figure 1 [file 13045_2014_58_MOESM1_ESM.gif]

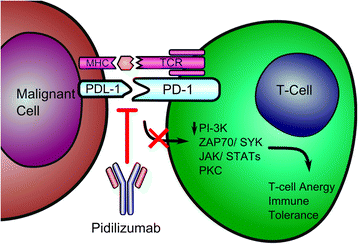

Supplement: Supplementary file 2 — Authors’ original file for figure 2 [file 13045_2014_58_MOESM2_ESM.gif]

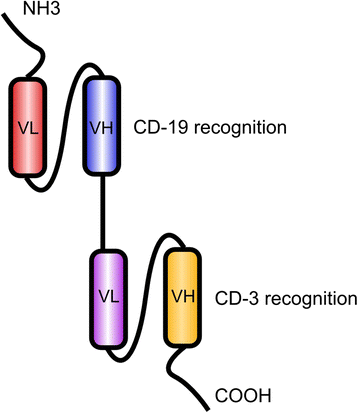

Supplement: Supplementary file 3 — Authors’ original file for figure 3 [file 13045_2014_58_MOESM3_ESM.gif]
